# Impact of Synthesis Parameters upon the Electronic Structure in PVD-Deposited Cd_*x*_Zn_1–*x*_O Composite Thin Films: An XPS-XANES Investigation

**DOI:** 10.1021/acsomega.4c00892

**Published:** 2024-02-14

**Authors:** Arkaprava Das, Ewa Partyka-Jankowska, Marcin Zając, Axel Hemberg, Carla Bittencourt

**Affiliations:** † Chimie des Interaction Plasma Surface, University of Mons, Place du Parc 23, 7000 Mons, Belgium; ‡ SOLARIS National Synchrotron Radiation Centre, Jagiellonian University, Czerwone Maki 98, 31-007 Krakow, Poland; § Materia Nova, Nicolas Copernic 3, 7000 Mons, Belgium

## Abstract

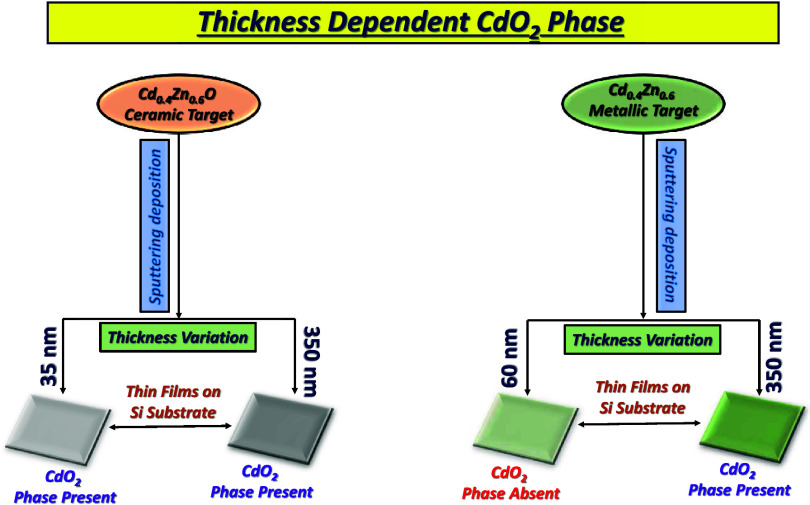

The impact of different synthesis parameters, such as thickness, postsynthesis annealing temperature, and oxygen gas flow rate, upon the electronic structure is discussed in detail in the present experimental investigation. X-ray photoelectron spectroscopy (XPS) and X-ray absorption near-edge structure (XANES) spectroscopy techniques are used to evaluate the surface electronic properties along with the presence and stability of the CdO_2_ surface oxide in Cd*_x_*Zn_1–*x*_O (*x* = 0.4) composite thin films. The thin films were synthesized with varying thicknesses using a Cd_0.4_Zn_0.6_O (CZO) ceramic and Cd_0.4_Zn_0.6_ (CZ) metallic targets and oxygen gas flow rates during magnetron sputtering. The Zn L_3,2_ edge and O K edge XANES spectra are affected by the oxygen gas flow rate. For the zero rate, an increase in intensity is observed in the Zn L_3,2_ edge, and notable changes occur in the overall spectral features of the O K edge. In the films synthesized in the presence of oxygen, highly probable O 2p → antibonding Zn 3d electronic transitions decrease the probability of the Zn 2p_1/2_ → antibonding Zn 3d electronic transition by filling the vacant antibonding Zn 3d states, leading to the reduction in overall intensity in the Zn L_3,2_ edge. Scanning electron microscopy reveals grain growth with increasing annealing temperature. The annealing induces orbital hybridization, generating new electronic states with higher transition probabilities and intensity enhancement in both Zn L_3,2_ and O K edges. The presence of the CdO_2_ surface phase is confirmed by analyzing the Cd 3d_5/2_ and O 1s XPS core levels. The CdO_2_ surface phase is observed in the films synthesized using the CZO target for all thicknesses, while the CZ target is only observed for higher thicknesses. Further postsynthesis annealing treatment results in the disappearance of the CdO_2_ phase. The CdO_2_ surface phase can be controlled by varying the film thickness and postsynthesis annealing temperature.

## 1 Introduction

There is much interest in semiconductor thin films due to their remarkable properties compared to bulk materials. As a result, extensive research has been conducted in this area to explore further and understand these materials’ potential applications.^1^ Previously, group III nitride thin films (GaN) were studied and reported as potential candidates for light-emitting diodes (LEDs), optical sensors, laser diodes, etc.^2,3^ However, their replacement becomes essential due to the costly synthesis of GaN thin films.^2^ As the optical characteristics and electronic structure of ZnO are quite like those of GaN, ZnO is accepted as an ideal replacement for GaN. ZnO is suitable for optoelectronic applications for large exciton binding energy (∼60 meV) and wide band gap values at room temperature (∼3.4 eV at 300 K).^4,5^ Synthesis of nanocomposites with CdO (band gap of ∼2.2 eV) and MgO (band gap of ∼7.8 eV) allows tuning the band gap in ZnO, which further renders them suitable to operate in the visible and ultraviolet regions of the electromagnetic spectrum, respectively.^6^ CdO has also recently attracted attention as a transparent conductor (TC) in optoelectronic devices due to its high electrical conductivity (>10^14^ S/cm) and high electron mobility (>100 cm^2^/V/s).^7−9^ Although CdO has superior electrical properties, a low band gap of 2.2 eV limits its applicability as a TC in optoelectronics. At room temperature and pressure under normal growth conditions, CdO and ZnO have rocksalt and wurtzite phases, respectively. With high Cd concentration, the rocksalt Cd_*x*_Zn_1–*x*_O nanocomposite can enhance the band gap while maintaining superior electrical properties for the TC in full-spectrum photovoltaics.^10,11^ Several growth mechanisms have been utilized under nonequilibrium conditions to achieve a single phase in the Cd_*x*_Zn_1–*x*_O composite in the midcomposition region. With *x* = 0.70, Detert et al. have reported a band gap of 2.6 eV and mobility of >60 cm^2^/V/s in the Cd_*x*_Zn_1–*x*_O composite in the rocksalt phase.^12^ The band gap is tuned from 3.3 to 1.8 eV up to 70% of Cd concentration in the wurtzite Cd_*x*_Zn_1–*x*_O composite thin film where the synthesis is performed using the metalorganic vapor deposition technique.^13,14^ Using the DC magnetron sputtering technique and varying Cd concentration up to 66%, the dominance of the hexagonal wurtzite phase is reported by Ma et al.^15^ With the pulsed filtered cathodic arc deposition method, wurtzite to rocksalt phase transformation (PT) is reported with *x* ∼ 0.7 Cd concentration in the Cd_*x*_Zn_1–*x*_O composite where the band gap is tuned from 3.2 to 1.8 eV.^16^ It is highly desirable yet challenging to attain a single-phase Cd_*x*_Zn_1–*x*_O composite within the midcomposition range, where the thermodynamic solubility of Cd in ZnO is < 2%.^17,18^ Hence, we aim to explore the electronic structure within this midcomposition region, where an optimal balance between optical and electrical properties can be achieved. Such an investigation could potentially lead to the development of efficient optoelectronic devices.

Comprehending material properties is pivotal in downsizing films to nanometer dimensions for thin film device applications. Among the various parameters, thickness is a critical factor that significantly impacts the structural, optical, and electrical properties. Therefore, it is imperative to conduct thorough investigations into the influence of thickness on microstructural, electrical, optical, and electronic properties to develop highly efficient optoelectronic devices. Despite being limited in number, existing reports explore the relationship between thickness and the optical, morphological, and optoelectronic properties of ZnO thin films.^19,20^ There is a shortage of comprehensive literature examining the electronic properties of Cd_*x*_Zn_1–*x*_O composite thin films in detail, particularly concerning their thickness dependency. Additionally, there is a lack of detailed analysis about Cd, Zn, and O surface states in such thin films, which have been deposited by using magnetron sputtering under varying synthesis parameters. Apart from film thickness, other synthesis parameters also influence the electronic structure properties of these heterostructural composite thin films. Therefore, we have varied three synthesis parameters, i.e., (1) thickness of the thin films, (2) oxygen gas flow rate during magnetron sputtering deposition, and (3) postsynthesis annealing temperature. The thin films were synthesized using a Cd_0.4_Zn_0.6_O (CZO) ceramic target and a Cd_0.4_Zn_0.6_ (CZ) metallic target. The information on surface phases and core-level orbital hybridizations for all the thin films is probed using X-ray photoelectron spectroscopy (XPS) and X-ray absorption near-edge structure spectroscopy (XANES). Only a few pieces of experimental evidence are available for the CdO_2_ phase in nanoparticles.^21^ In the present thin film system, we are able to find the traces of the CdO_2_ surface phase from XPS observation. Therefore, the impact of thickness and postsynthesis annealing temperature upon the CdO_2_ phase has also been investigated in detail. The intensity variation in the Zn L_3,2_ edge and change in overall spectral features in the O K edge XANES spectra are explained from the perspective of core-level electronic transitions.

## 2 Experimental Methods

The thin films were synthesized using the magnetron sputtering technique, and deposition was performed on the Si wafer substrate. Here, we performed thin film synthesis using two separate targets. The Cd_0.4_Zn_0.6_O (CZO) ceramic target (99.99%, 2″ diameter, and 3 mm thick with the copper backing plate, Jiangyin Maideli Advanced Materials Co., Ltd., China) has been used with a radio frequency (RF) generator operated at 100 W power and 13.56 MHz frequency. The Cd_0.4_Zn_0.6_ (CZ) metallic target (99.99%, 2 in. diameter, and 3 mm thick, Jiangyin Maideli Advanced Materials Co., Ltd., China) was used with a pulsed power supply operated at 100 W power and 250 kHz frequency. The distance between the cathode and Si substrate was fixed for both targets at 10 cm. The deposition was performed at 7 × 10^–3^ Torr working pressure for both targets. The total gas flow was 10 standard cubic centimeters per minute (SCCM). With the CZO ceramic target, the oxygen gas flow rate was kept at zero. The thin film deposition was performed with four different oxygen gas flow rates with the CZ metallic target. The ratio between Ar and O gas flow rates is 8:2, 6:4, 4:6, and 0:10. The thickness calibration was performed using a DEKTAK profilometer (Bruker). For the CZO target, the desired thickness of ∼350 nm was achieved with 20 min of deposition. Further, thin films of thicknesses 35, 125, and 450 nm were also prepared with the CZO target with proper deposition duration. Therefore, the deposition rate was ∼0.3 nm/s. The required deposition time was ∼49 min to fetch the desired thickness of ∼350 nm for the CZ target. Therefore, the deposition rate was ∼0.12 nm/s with a pulsed power supply. With this deposition rate, 7.5 min was required to synthesize a ∼60 nm-thick film. For each oxygen gas flow rate, we calibrated the thickness. The deposition rate is not significantly affected by the oxygen gas flow rate. The deposition rate with the RF generator for the ceramic target was almost 3 times that with a pulsed generator for the metallic target. The postsynthesis annealing was performed in a tubular furnace with a flowing oxygen gas environment. X-ray diffraction (XRD) measurements were performed with a Bruker high-resolution X-ray diffractometer system using a Cu Kα beam. Surface oxidation states were investigated with an ESCA-5000 (Physical Electronics) Versa Probe system using an Al Kα (1486.7 eV) beam attached to a 124 mm hemispherical electron analyzer. X-ray absorption near-edge spectroscopic (XANES) measurements have been performed for Zn L_2,3_ and O K edges in total electron yield (TEY) detection mode at the PIRX beamline^22^ in the SOLARIS synchrotron, Poland.^23^ Measurements were performed with unpolarized light and with a perpendicular incidence upon the sample surface.

## 3 Results and Discussion

### 3.1 X-ray Diffraction Pattern

**Figure 1:**
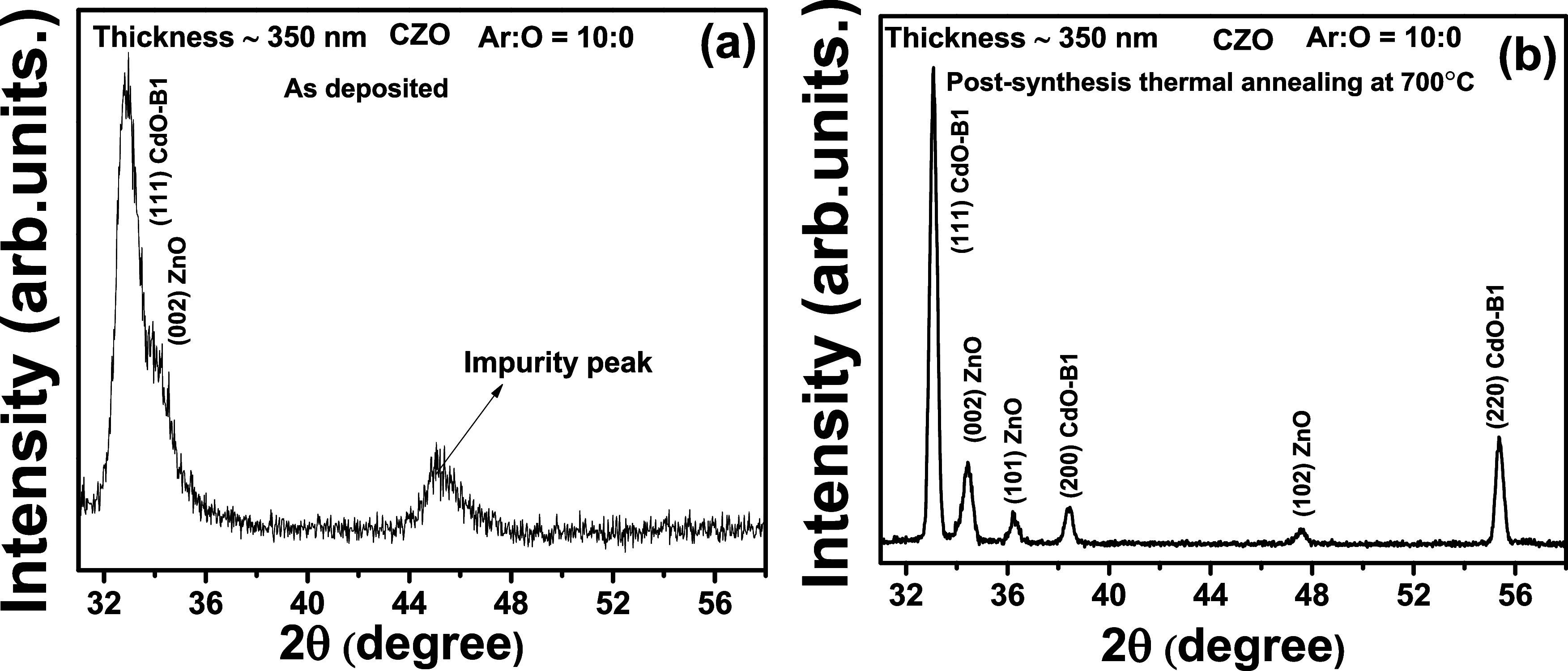
(a, b) X-ray diffraction patterns for as-deposited (a) and thermally annealed (b) thin film-synthesized CZO ceramic target with the same thickness (∼350 nm).

Figure 1a shows the XRD pattern for the as-deposited thin film with a thickness of 350 nm deposited using the CZO ceramic target with 10:0 ratio of Ar to O gas flow. The broad, intense reflection at 32.9° is the signature of the rocksalt B1 phase for CdO. The reflection at 34.1° convoluted with the CdO B1 phase reflection indicates the wurtzite ZnO phase, whereas the reflection observed at 45.1° possibly arises from some impurity. Therefore, the XRD pattern of the as-deposited thin film provides a signature of a poor crystalline thin film. The crystalline quality of the film improved after annealing at 700 °C in an oxygen gas environment (Figure 1b). The presence of ZnO and CdO phases is evident, and there is no trace of any extra reflection. At 33.0, 38.5, and 55.4°, (111), (200), and (220) reflections are observed for the CdO rocksalt phase (B1) (space group *Fm*3̅*m*). The reflections corresponding to the hexagonal wurtzite ZnO phase (space group *P*6_3*mc*) for (002), (101), and (102) Bragg peaks are observed at 34.3, 36.3, and 47.6°, respectively. For the annealed thin film, the reflections are identical with the wurtzite ZnO and rocksalt CdO phases. There are no extra reflections, which may correspond to the CdO_2_ surface phase. Therefore, the XRD technique cannot detect the presence of the CdO_2_ surface phase, which has been successfully detected with the XPS technique. The amount of the CdO_2_ phase is quite minimal and not within the detection limit of XRD. The CdO_2_ phase has only been reported for the nanoparticles, not for the thin films, to the best of our knowledge. Bragg’s reflections of the CdO_2_ phase (space group *Pa*3̅) in the nanoparticle system are situated at 33.7, 48, 57.4, and 60.1° for (111), (200), (220), and (311) Bragg peaks, respectively.^21^ As per the ICDD database (PDF No. 391221), the Bragg reflection peaks appear at 29.1° (111), 33.7° (200), 37.9° (210), 41.7° (211), 48.4° (220), 57.6° (311), and 60.3° (222) for the CdO_2_ phase. These reflections are not observed in the XRD patterns shown in Figure 1a,b.

### 3.2 Grain Growth due to Postsynthesis Annealing Treatment

**Figure 2:**
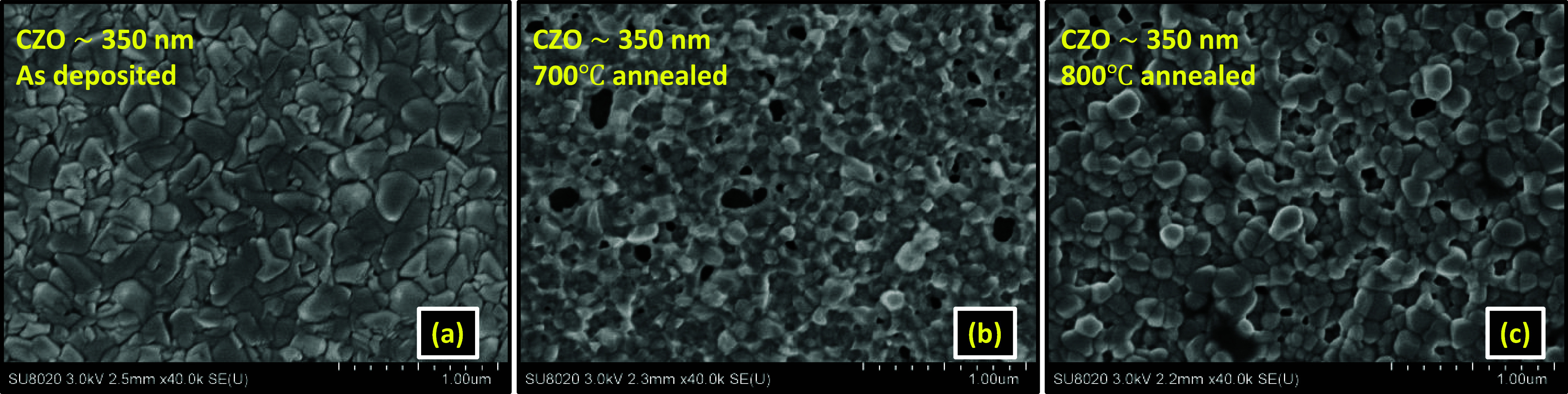
SEM micrographs for Cd_0.4_Zn_0.6_O target-deposited thin films. (a) As-deposited thin film with ∼350 nm thickness and (b) 700 °C-annealed and (c) 800 °C-annealed thin film.

Figure 2a–c shows the scanning electron microscopic images in the 1 μm scale for the thin films synthesized from the CZO target. In Figure 2a, the as-deposited thin film with ∼350 nm thickness indicates a compact morphology without any crack or presence of any dust particle. Figure 2b,c shows the morphology for 700 and 800 °C-annealed thin films. Grain growth due to postsynthesis thermal treatment is visible from SEM micrographs. Out of the cluster migration and Ostwald ripening growth processes, in the present circumstances, the second process is dominating as the annealing temperature is greater than 500 °C. In that process, a positive interfacial energy appears at the grain boundary due to modification in the chemical potential at the junction between the nanocrystallites.^24^ The interfacial energy further accelerates grain growth, and grains with enhanced curvature at the grain boundary region are generated with thermal treatment. The spherical grains are visible in Figure 2b,c. For 800 °C annealing, larger spherical grains appear in the image with better connectivity. The observed grain growth agrees with the enhanced intensity in O K and Zn L_3,2_ edge XANES spectra in Figures 10a and 14a. This correlation is discussed in the following sections.

### 3.3 Evidence of the Thickness-Dependent CdO_2_ Surface Phase from X-ray Photoelectron Spectroscopy

**Figure 3:**
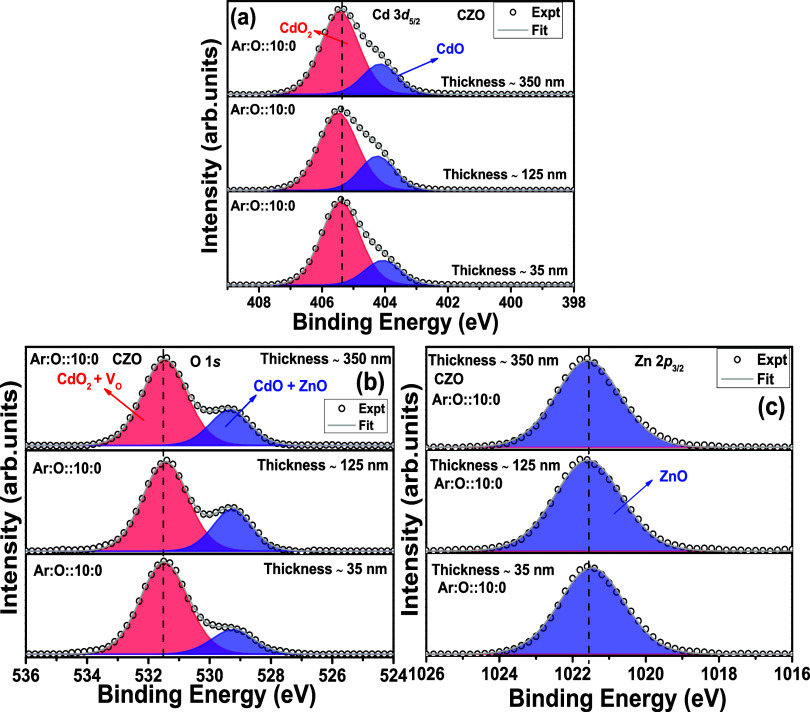
(a–c) Cd 3d, O 1s, and Zn 2p edge XPS spectra for Cd_0.4_Zn_0.6_O ceramic target-deposited thin films with different thicknesses (∼35, 125, 350 nm).

Figure 3a–c shows the Cd 3d_5/2_, O 1s, and Zn 2p_3/2_ peaks in XPS spectra for the thin films with different thicknesses and deposited using the CZO ceramic target. All XPS spectra have been calibrated with a 284.6 eV C 1s peak. The peak fitting is performed with CASA software using mixed Gaussian (70%) and Lorentzian (30%) (GL) functions. For background removal, the Shirley background has been used. Cd 3d and Zn 2p spectra have an apparent doublet, i.e., Cd 3d_3/2_ and Cd 3d_5/2_ and Zn 2p_3/2_ and Zn 2p_1/2_, respectively. Figure 3a,c shows the Cd 3d_5/2_ and Zn 2*p*_3/2_ peaks. In Figure 3a, from the deconvolution, a strong presence of CdO (404.1 eV) and CdO_2_ (405.4 eV) phases is observed. There is no shift in binding energy (BE) values with increasing thickness for CdO and CdO_2_ phases. Therefore, there is no thickness dependence for the thin films deposited using ceramic CZO targets for the robust CdO_2_ surface phase. In Figure 3b, the O 1s peak is deconvoluted into two broad peaks for all three thin films. The peak at the lower BE side (529.2 eV) corresponds to the Cd–O and Zn–O bonds. The broad peak at the higher BE side (531.5 eV) suggests the presence of CdO_2_ and possible oxygen vacancies (V_O_).^24,25^ The presence of the CdO_2_ phase at a higher BE side compared to the CdO phase is well reported by Piper et al.^26^ The CdO_2_ phase is reported as a surface phase. Therefore, the related X-ray diffraction pattern for the CdO2 surface phase in the thin film is not reported in the literature, to the best of our knowledge. The Zn 2p_3/2_ peak in Figure 2c at 1021.5 eV is the signature of Zn–O bonds, i.e., Zn^2+^ oxidation state.^27^ The O 1s and Zn 2p_3/2_ peaks do not show any thickness dependence, as the peak position remains at the same BE value for all thicknesses.

**Figure 4:**
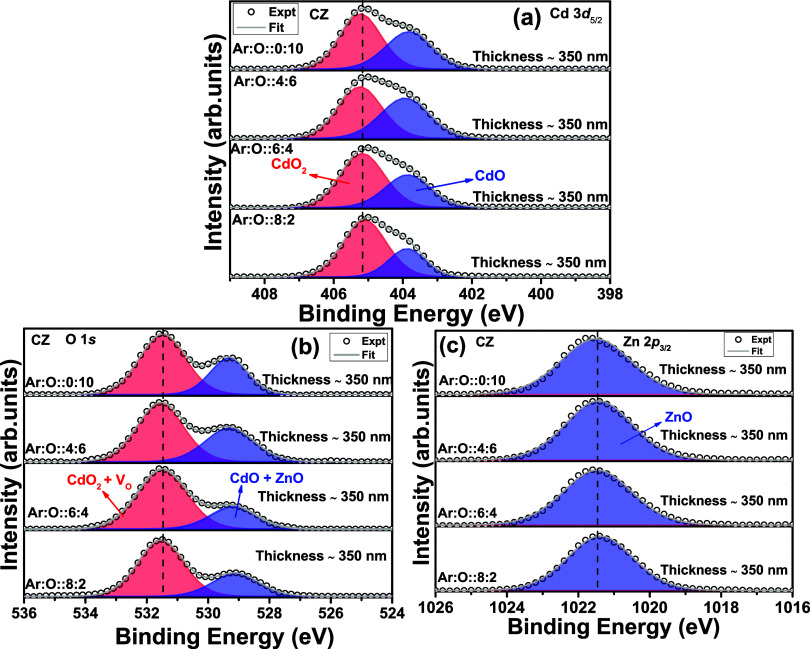
(a–c) Cd 3d, O 1s, and Zn 2p edge XPS spectra for Cd_0.4_Zn_0.6_ metallic target-deposited thin films (∼350 nm thickness) with different oxygen gas flow rates.

In Figure 4a–c, Cd 3d_5/2_, O 1s, and Zn 2p_3/2_ peaks are shown for CZ metallic target-deposited thin films where the O gas flow rate increases. The oxygen gas flow is varied from 2 SCCM to 10 SCCM, keeping the film thickness constant at ∼350 nm. The XPS spectra are stacked in Figure 4 from bottom to top with increasing oxygen gas flow rate. The Ar and O gas flow rates are listed in Figure 4. In Figure 4a, the presence of the CdO_2_ phase (401.2 eV) is evident from the deconvolution of the Cd 3d_5/2_ peak. Therefore, if the thickness is constant and the oxygen gas flow rate increases gradually, the CdO_2_ phase is not influenced. In Figure 4b, the deconvolution of the O 1s peak is the same as in Figure 3b. However, the peak area corresponding to Cd^2+^ and Zn^2+^ at 529.3 eV increases with increasing oxygen partial gas pressure. The peak area in Figure 4a corresponding to Cd^2+^ also increases, providing a concurring observation between the Cd 3d_5/2_ and the O 1s peak. The Zn 2p_3/2_ edge in Figure 4c does not indicate any notable change with increasing oxygen gas flow rate. This discussion confirms direct evidence that the oxygen gas flow rate improves the presence of the CdO phase and not the CdO_2_ or ZnO phases.

**Figure 5:**
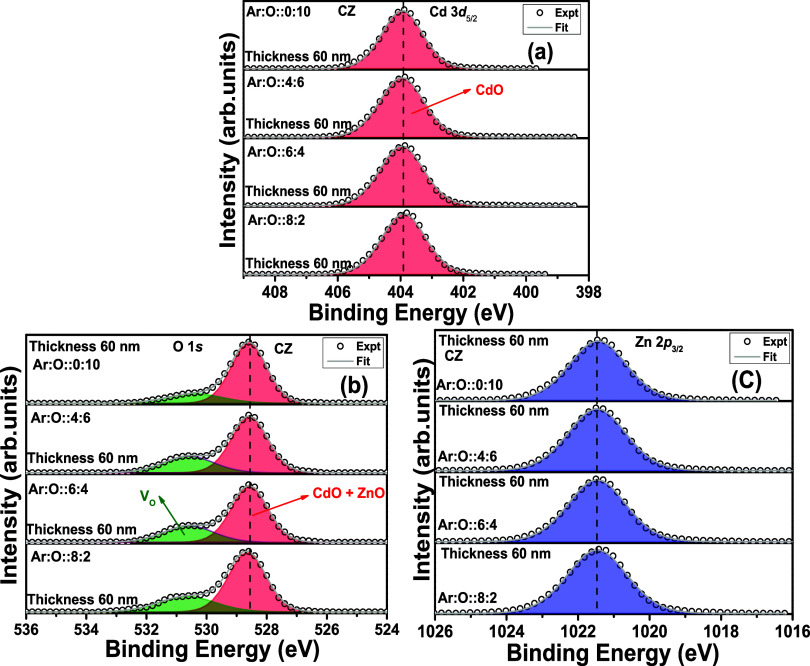
(a–c) Cd 3d, O 1s, and Zn 2p edge XPS spectra for Cd_0.4_Zn_0.6_ metallic target-deposited thin films (∼60 nm thickness) with different oxygen gas flow rates.

Figure 5a–c shows the Cd 3d_5/2_, O 1s, and Zn 2p_3/2_ edge XPS spectra with CZ target-deposited thin films with the thickness fixed at ∼60 nm. The oxygen gas flow rate varies from 2 to 10 SCCM, keeping the total gas flow of (Ar + O) fixed at 10 SCCM. The peak fitting of the Cd 3d_5/2_ peak in Figure 5a shows a notable change compared to those in Figures 3a and 4a. In the case of ∼350 nm thickness with the CZ target and for all thicknesses with the CZO target, the Cd 3d_5/2_ peak has shown an asymmetry in the peak. Due to that asymmetry, further deconvolution is performed considering two GL functions. However, in the case of a CZ target with ∼60 nm thickness, the peak corresponding to the Cd 3d_5/2_ peak is symmetric and can be fitted using one GL function without any further deconvolution. The position of this symmetric Cd 3d_5/2_ peak is at 403.9 eV, which is close to the position of the Cd^2+^ oxidation state. Therefore, in a 60 nm-thick film, only the CdO phase is quite clear. This further proves the thickness dependence of the CdO_2_ surface phase. Therefore, we can enunciate that higher thickness is favorable for CdO_2_ surface formation and lower thickness does not support the same. However, we cannot consider 60 nm thickness as the cutoff thickness for CdO_2_ phase formation with the metallic CZ sputtering target. For the CZO sputtering target, the CdO_2_ phase is present even with ∼35 nm thickness. Therefore, CdO_2_ phase formation does not solely depend on thickness parameters. Instead, it has an implicit dependence upon thickness. It is worth mentioning that CdO_2_ phase formation has no explicit or implicit dependence upon the oxygen gas flow rate during synthesis. In Figure 5b, the overall O 1s XPS spectra are significantly changed compared to Figures 3b and 4b. The relative peak area corresponding to the Cd–O and Zn–O states at 528.6 eV in the BE scale has increased in Figure 5b compared to those in Figures 3b and 4b. The peak centered at 530.7 eV indicates only the presence of V_O_ in Figure 5b. The same peak in Figures 3b and 4b with a larger area corresponds to the combined presence of CdO_2_ and V_O_. However, in Figure 5b, the significant deterioration in the intensity of this peak indicates the absence of the CdO_2_ surface phase. With increasing oxygen partial gas pressure, the area of this V_O_ peak is further decreased. More oxygen atoms during the sputtering process result in fewer V_O_ defects in the thin film than others with the same thickness. Therefore, the Cd 3d_5/2_ and O 1s peaks provide a concurring observation regarding the absence of a CdO_2_ surface phase for 60 nm-thick films with a CZ sputtering target. The Zn 2p_3/2_ peak in Figure 5c remains unchanged with increasing oxygen gas flow rate.

**Figure 6:**
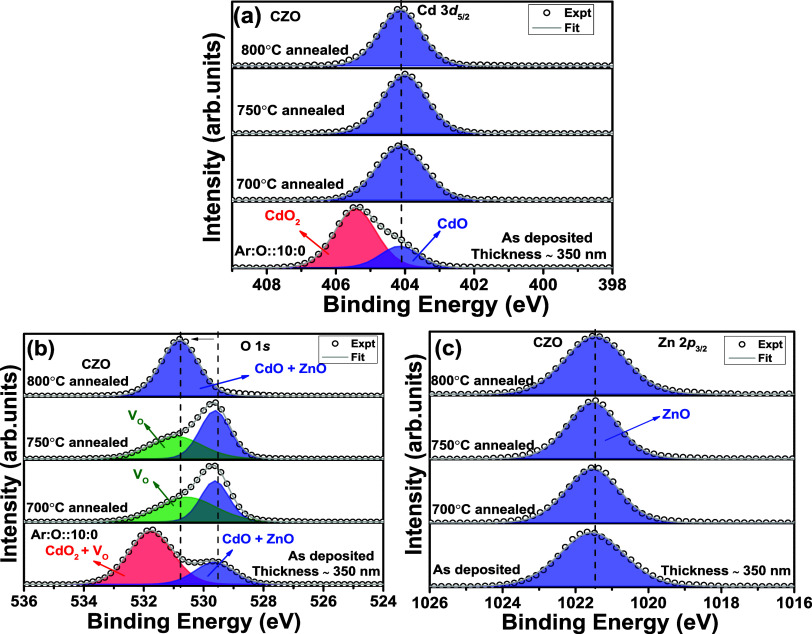
(a–c) Cd 3d, O 1s, and Zn 2p edge XPS spectra for Cd_0.4_Zn_0.6_O ceramic target-deposited thin films (∼350 nm thickness) with postsynthesis annealing treatment.

To check the impact of postsynthesis thermal annealing upon the CdO_2_ surface phase, we have performed thermal annealing of the 350 nm-thick film deposited from the CZO target. The annealing was performed at 700, 750, and 800 °C for 1 h in a flowing oxygen ambience in a tubular furnace. Figure 6a–c shows the Cd 3d_5/2_, O 1s, and Zn 2p_3/2_ peak XPS spectra of as-deposited and annealed thin films at three different temperatures, respectively. The Cd 3d_5/2_ peak for the as-deposited spectra is the same as that in Figure 3a. Figure 6a shows that the CdO_2_ phase is absent from all three annealed thin films. Only the Cd^2+^ state is present in the annealed thin films.^24^ This further allows us to conclude that the CdO_2_ surface phase explicitly depends on the postsynthesis thermal annealing temperature. In Figure 6b, the O 1s peak suffered a significant change with thermal annealing compared to the as-deposited thin films. The peak area at the higher BE side decreases with increasing thermal annealing temperature. For the 700 °C-annealed thin film, the deconvoluted peak at the higher BE side is only anticipated to correspond to V_O_. The V_O_ peak is absent for the 800 °C-annealed thin film, and the peak corresponding to combined Cd–O and Zn–O bonds has shifted to the higher BE side. Annealing with a longer duration in flowing oxygen gas has provided sufficient time for atoms in the thin film to readjust their position to fetch the system to a more stable condition. This might be the reason for shifting the peak to a higher BE side. The flowing oxygen gas atoms at high temperatures might have reduced the V_O_ density significantly, which leads to the absence of deconvolution related to the same. The Zn 2p_3/2_ peak XPS spectra in Figure 6c are unchanged with postsynthesis annealing treatment. Therefore, out of three synthesis parameters, i.e., thickness, oxygen gas partial pressure, and postsynthesis annealing, the presence and stability of the CdO_2_ surface phase depend upon the thickness and postsynthesis annealing treatment. The annealing at high temperatures leads to evaporation of the CdO_2_ surface phase. Therefore, apart from film thickness, postsynthesis annealing treatment also determines the possible existence of the CdO_2_ surface phase.

### 3.4 Impact of Film Thickness, Oxygen Partial Pressure, and Annealing Temperature upon Zn L_3,2_ and O K Edges

#### 3.4.1 Zn L_3,2_ Edge

**Figure 7:**
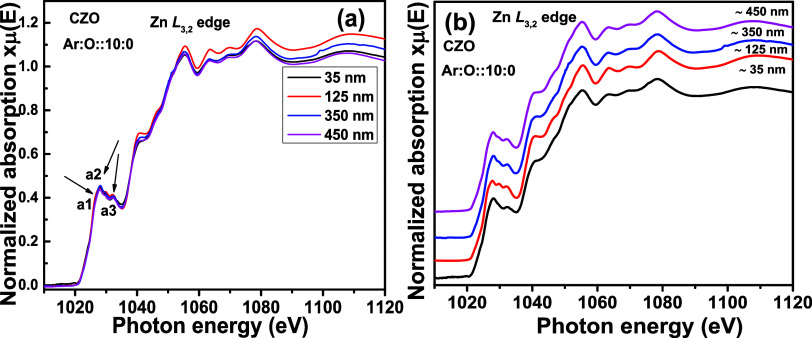
Zn L_3,2_ edge XANES spectra for Cd_0.4_Zn_0.6_O (CZO) sputtering target-deposited thin films with different thicknesses. In (a), all normalized XANES spectra are overlapped, and in (b), they are stack-plotted.

Figure 7a shows the pre- and postedge-corrected normalized Zn L_3,2_ edges for CZO thin films with different thicknesses. All measurements are performed with unpolarized X-rays. The pre-edge normalization is performed by fitting a straight line before the absorption edge (below 1015 eV). Further postedge normalization is performed by fixing the intensity of the spectra to 1 at 1080 eV.^28^ For four different thicknesses, i.e., 35, 125, 350, and 450 nm, Zn L_3,2_ edges are recorded in TEY mode. In Figure 7b, the same is stack-plotted. The main absorption begins around 1020 eV. The a1 feature (1027 eV) (Figure 7a) in the main absorption edge is generated due to the electronic transition from filled Zn 2p to vacant Zn 4s states.^29,30^ There is also the finite possibility of a Zn 2p → Zn 3d electronic transition. The 4s orbital is less localized than the 3d orbital. Therefore, the electronic transition from the Zn 2p orbital is more probable in 3d orbitals. Apart from the less localized character of d orbitals, p to d core orbital dipole-allowed transitions are dominating in transition metals owing to higher cross-section and occupation probability of d orbitals.^31^ Next, a2 and a3 features at 1028.1 and 1032.3 eV are the consequence of the Zn 2p_1/2_ → 3d antibonding transition and Zn 2p → 4s transitions, respectively.^31^ Apart from this, the density of states at ∼1041 and ∼1047 eV is attributed to the transitions from Zn 2p to hybridized O 2p–Zn 4d(t_2g_) and O 2p–Zn 4d(e_g_) orbitals, respectively.^30^ The a1, a2, and a3 features do not change with the changing thickness of the thin film, which is evident as the measurements have been performed with surface-sensitive TEY mode. Therefore, the increasing thickness does not generate any Zn-related secondary phase. Beyond 1040 eV, no thickness-dependent change in the oscillations is observed. More detailed discussion for Zn L_3,2_ edges requires a symmetry-projected band structure calculation, which is beyond the scope of the article. However, our experimental spectra are in good agreement with the simulated Zn L_3,2_ edge for the wurtzite ZnO structure by Mizoguchi et al.^32^ and Gilbert et al.^33^ Therefore, the photoabsorbing Zn atom is residing in a fourfold coordination with the O atom in the thin film. This is also in agreement with the phase identification from the XRD pattern in Figure 1b.

**Figure 8:**
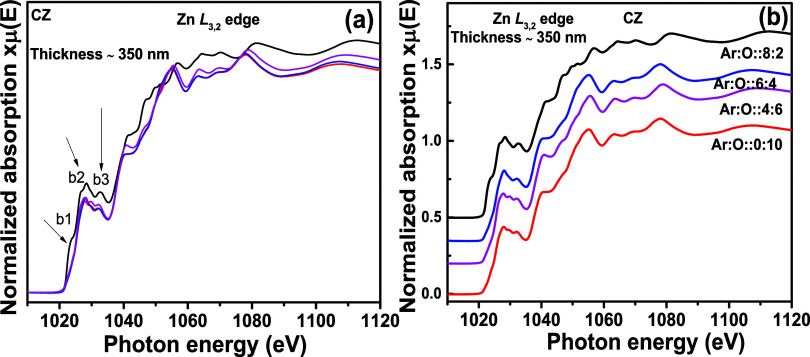
Zn L_3,2_ edge XANES spectra for Cd_0.4_Zn_0.6_ (CZ) sputtering target-deposited thin films with ∼350 nm thickness and different oxygen gas flow rates. In (a), all normalized XANES spectra are overlapped, and in (b), the same are stack-plotted.

Figure 8a shows the Zn L_3,2_ edge spectra for CZ thin films at different oxygen gas flow rates during the sputtering synthesis. In Figure 8b, these spectra are stacked and plotted for better clarity in observation. In Figure 8a, the overlapped spectra indicate higher spectra for a thin film with 2 SCCM oxygen gas flow during synthesis. Except for this one, the remaining three spectra with increasing oxygen partial pressure remain unchanged in the absorption region. The intensity enhancement in the b1 feature (black spectra) indicates metallic Zn behavior. In the presence of metallic Zn, the probability of electronic transitions from Zn 2p to vacant Zn 4s states is higher than when it remains in the oxide form. The transition from O 2p to empty Zn 4s states is highly probable in the Zn–O bond. Therefore, the density vacancies in unfilled Zn 4s states are reduced in the presence of oxygen, and the probability for Zn 2p → Zn 4s transitions is also decreased.^31^ This explanation is valid for b2 and b3 features, where similar intensity enhancement is observed without Zn–O bonds. The b2 feature is caused by Zn 2p_1/2_ → Zn 3d antibonding transitions prominent in metallic Zn.^31^ When Zn–O bonds are present in the lattice, O 2p → Zn 3d electronic transitions are also possible. These transitions are anticipated to fill the vacant Zn 3d antibonding states and reduce the probability of Zn 2p_1/2_ → Zn 3d electronic transitions. Therefore, in metallic Zn, only the Zn core orbital-related transition is strong and results in an intensity enhancement compared to other oxide thin films. Here, it is essential to note that the thickness for all thin films is the same, i.e., ∼350 nm in Figure 8a,b. The intensity variation occurs due to the difference in the electronic transition probability caused by different oxygen gas flow rates.

**Figure 9:**
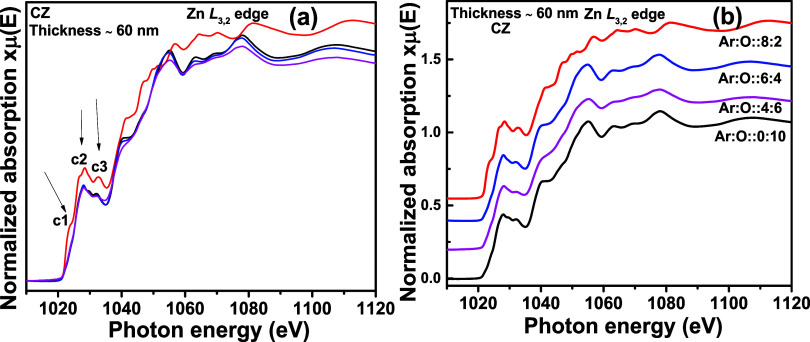
Zn L_3,2_ edge XANES spectra for Cd_0.4_Zn_0.6_ (CZ) sputtering target-deposited thin films with ∼60 nm thickness and different oxygen gas flow rates. In (a), all normalized XANES spectra are overlapped, and in (b), the same are stack-plotted.

Figure 9a portrays the Zn L_3,2_ edge spectra for CZ thin films at different oxygen partial pressures for 60 nm thin films. The main absorption features for c1, c2, and c3 are similar, as shown in Figures 7a and 8a. With 60 nm thickness, we observe similar intensity enhancement in c1, c2, and c3 features compared with 350 nm thickness. Therefore, the Zn L_3,2_ edge spectra are not sensitive to the thickness variation. As the measurements are done in TEY mode, the photoelectrons generated from a few nm (∼10 nm) depth from the thin film surface are measured. Therefore, 60 and 350 nm-thick films virtually behave as bulk materials for XAS spectroscopy.

**Figure 10:**
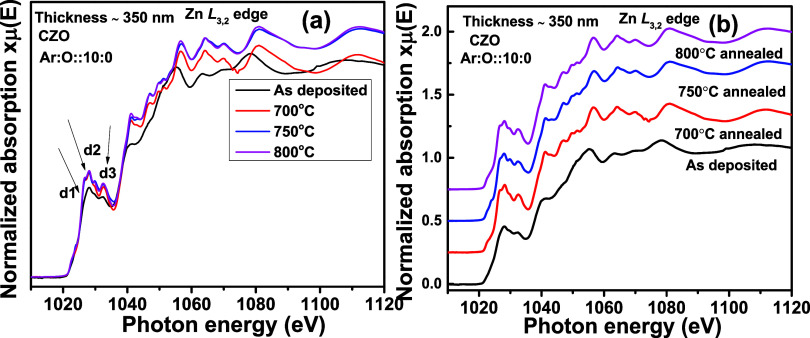
Zn L_3,2_ edge XANES spectra for Cd_0.4_Zn_0.6_ (CZO) sputtering target-deposited thin films with ∼350 nm thickness and different postsynthesis thermal annealing treatments. In (a), all normalized XANES spectra are overlapped, and in (b), the same are stack-plotted.

Figure 10a shows the Zn L_3,2_ edge spectra for the thin films synthesized from the CZO sputtering target with a thickness of ∼350 nm. The same are stack-plotted in Figure 10b. The thin films are annealed at 700, 750, and 800 °C, in a flowing oxygen ambience. For comparison, we have included the Zn L_3,2_ edge spectra for the as-deposited thin film shown in Figure 7a. The d1, d2, and d3 features are similar to the a1, a2, and a3 features of Figure 7a. There is an overall intensity enhancement in annealed thin films compared to the as-deposited ones. From SEM micrographs in Figure 2, it is quite clear that crystallinity has been enhanced with visible grain growth. The core-level electronic transition probability due to X-ray absorption is higher in defect-free crystalline thin films. Therefore, the dipole-allowed electronic transitions are stronger, enhancing intensity. In the as-deposited thin film, there is a possibility of relaxation in dipole-allowed core-level transitions due to a greater number of defects. Such transitions may arise out of phase oscillations and further produce destructive interference within the multiple scattering signals. This may explain the lower intensity of the as-deposited thin film.

#### 3.4.2 O K Edge

**Figure 11:**
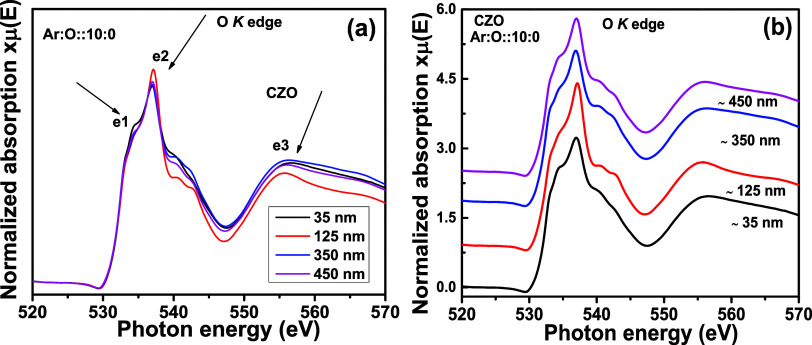
O K edge XANES spectra for Cd_0.4_Zn_0.6_O (CZO) sputtering target-deposited thin films with different thicknesses. In (a), all normalized XANES spectra are overlapped, and in (b), the same are stack-plotted.

Figure 11a shows the O K edges for CZO thin films with four different thicknesses (35, 125, 350, 450 nm). In Figure 11b, the same are stacked. No significant change in the intensity or any of the e1, e1, and e3 absorption features is observed with thickness variation. The strong absorption between 530 and 537 eV corresponds to the electronic transitions between the O 1s to hybridized O 2p and Zn 4s states. Mainly, the e1 feature corresponds to the transitions from O 1s to nonlocalized Zn 4s states, resulting in a broad feature. The sharp e2 feature arises from the electronic transition from the O 1s core level to localized and dipole-allowed O 2p states.^34,35^ The absorption features observed within 539–550 eV are mainly attributed to the transition from the O 1s to the hybridized O 2p and Zn 4d orbitals. The features above 550 eV, i.e., the e3 feature, are also the results of the transition from O 1s to hybridized O 2p and Zn 4d orbitals.^34,35^ Therefore, like the Zn L_3,2_ edge, the O K edge also indicates a concurring observation and does not show any significant change with thickness variation. The O K edge spectra for wurtzite ZnO and rocksalt CdO structures are reported by Frati et al.^36^ The K edge spectra for these two structures are different. In the CdO structure, Cd is octahedrally coordinated with the O atom, whereas in the ZnO wurtzite structure, Zn is tetrahedrally coordinated, giving rise to different O 1s → 2p soft X-ray absorption spectra.^37^ However, in our present scenario, the O K edge spectra are mainly similar to the wurtzite ZnO structure.

**Figure 12:**
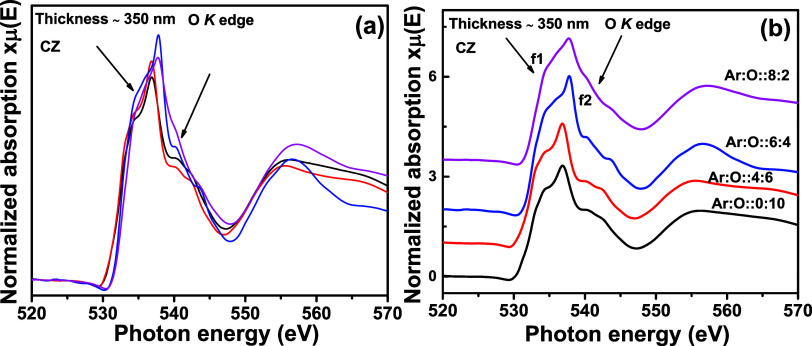
O K edge XANES spectra for Cd_0.4_Zn_0.6_ (CZ) sputtering target-deposited thin films with a thickness of ∼350 nm and different oxygen gas flow rates. In (a), all normalized XANES spectra are overlapped, and in (b), the same are stack-plotted.

Figure 12a shows the O K edges for a CZ thin film (∼350 nm thickness) with different oxygen partial pressures during sputtering. The main features are described in the previous section. In Figure 12b, in the stack plot, we can observe a change in f1 and f2 features at 534.3 and 540 eV, respectively, where the oxygen partial pressure during synthesis is minimal compared to other thin films. The f1 and f2 features have broadened. Due to less oxygen with 2 SCCM flow rate during synthesis, the density of states for O 2p core states is also minimal, which does not allow enough orbital hybridization between Zn 4d and O 2p core states. Therefore, mainly, the electronic transitions from the O 1s level to the nonlocalized Zn 4s level take place, resulting in a broad f1 feature. Such broadening of the O K edge is reported by Krishnamurthy et al. for Co-doped ZnO thin films where the presence of V_O_ has been considered the prime reason for such broadening.^34^ Similarly, the f2 feature also broadens due to deterioration in Zn 4d and O 2p orbital overlapping and subsequent low transition probability to those hybridized states with minimal Zn–O bonds. A right hand-side shift in the absorption edge is observed, owing to the metallic nature of the thin film. The presence of V_O_ alters the binding energies of the nearby surrounding shells, which has caused the shift in the onset of the O K edge toward high photon energy.^34^ Demchenko et al. have calculated the different O K spectra for CdO with different amounts of V_O_.^38^ They showed that increasing the number of V_O_, the difference between the ideal CdO structure and defected CdO structure increases. Therefore, theoretically, the impact of V_O_ in the overall O K edge spectra for such a transition metal oxide system is well established.

**Figure 13:**
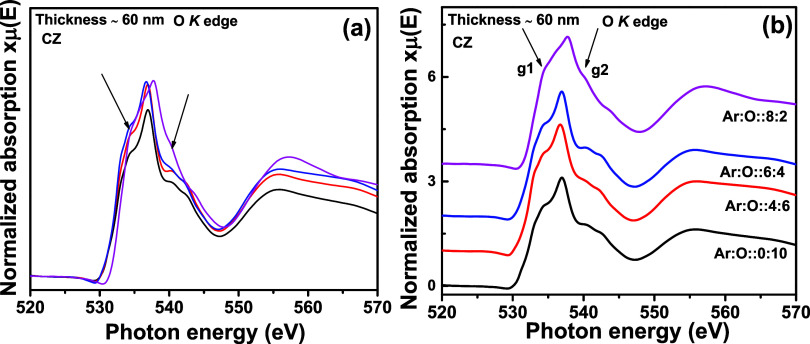
O K edge XANES spectra for Cd_0.4_Zn_0.6_ (CZ) sputtering target-deposited thin films with a thickness of ∼60 nm and different oxygen gas flow rates. In (a), all normalized XANES spectra are overlapped, and in (b), the same are stack-plotted.

Figure 13a portrays O K edge spectra for 60 nm-thick CZ thin films, with different oxygen partial pressures used during sputtering synthesis. In Figure 13b, similar to the 350 nm-thick film, no significant change is observed in the O K edge spectra except for g1 and g2 features at 534 and 540 eV, respectively, where the O partial pressure is minimal. Therefore, 350 and 60 nm thin films provide a concurring observation. O K edge features of the O are more impacted by oxygen partial pressure used during synthesis rather than the thickness of the film.

**Figure 14:**
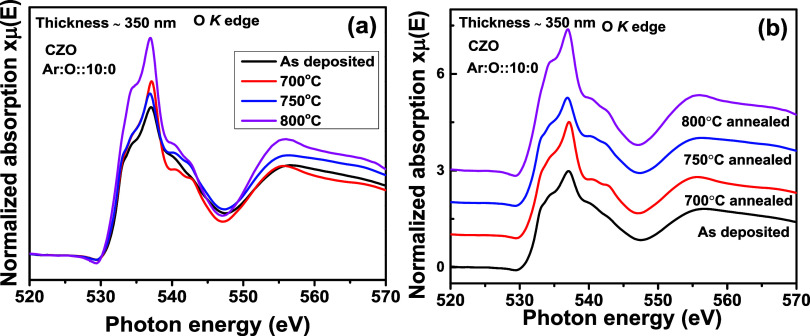
O K edge XANES spectra for Cd_0.4_Zn_0.6_ (CZO) sputtering target-deposited thin films with ∼350 nm thickness and different postsynthesis thermal annealing treatments. In (a), all normalized XANES spectra are overlapped, and in (b), the same are stack-plotted.

Figure 14a shows O K edge spectra for CZO thin films (thickness of ∼350 nm) with postsynthesis annealing treatment at 700, 750, and 800 °C. The spectrum for the as-deposited sample is collected from Figure 11a. In Figure 14b, the stacked spectra are plotted. From Figure 14a, we observed that due to thermal annealing, the intensity is enhanced compared to that of deposited thin films. The intensity is highest for thermal annealing at 800 °C. As the thermal annealing is performed in a flowing oxygen ambience, defects such as V_O_ decrease, making the thin film surface defect-free. This reduction in V_O_ is the possible reason for the gradually increasing intensity of the K edge spectra. Due to the improvement in crystallinity, the intensity of the Zn L_3,2_ edge also increases compared to the as-deposited thin film in Figure 10a. However, a gradual increment in intensity, like O K edge spectra, is not observed in the Zn L_3,2_ edge. Therefore, this indicates that V_O_ defects impact the K of the O K edge more than the Zn L_3,2_ edges.

## 4 Conclusions

We have synthesized Cd_*x*_Zn_1–*x*_O nanocomposite thin films on a Si wafer substrate using plasma sputtering with a Cd_0.4_Zn_0.6_O (CZO) ceramic and Cd_0.4_Zn_0.6_ (CZ) metallic sputtering targets. The CdO_2_ surface phase is present in all thin films for ceramic target-deposited thin films, irrespective of their thickness. For metallic target-deposited thin films, the CdO_2_ surface phase is observed only in films with higher thickness (∼350 nm) and not with lower thickness (∼60 nm). The thermal annealing at a higher temperature enhances the intensity of features in both Zn L_3,2_ and O K edge spectra. With metallic CZ target-deposited films, Zn L_3,2_ edge intensity was enhanced with the least oxygen gas flow. The absence of Zn–O bonds in that thin film enhances the probability of Zn 2p → 3d antibonding electronic transitions, which further results in an intensity enhancement of absorption spectra. The deconvolution of 3d_5/2_ and O 1s peaks in XPS spectra provides evidence of the CdO_2_ phase at a binding energy side higher than that of the CdO phase. Apart from this thickness sensitivity of the CdO_2_ phase, it also depends on the postsynthesis thermal annealing treatment. For the thin films synthesized with the ceramic target deposited, the CdO_2_ phase after the thermal annealing treatment disappeared. We have performed a thorough study regarding the impact of three synthesis parameters, i.e., thickness, thermal annealing, and oxygen gas flow rate, on electronic properties, which can help in fabricating an efficient optoelectronic device in the future.
